# Higher Nevus Count Exhibits a Distinct DNA Methylation Signature in Healthy Human Skin: Implications for Melanoma

**DOI:** 10.1016/j.jid.2016.11.029

**Published:** 2017-04

**Authors:** Leonie Roos, Johanna K. Sandling, Christopher G. Bell, Daniel Glass, Massimo Mangino, Tim D. Spector, Panos Deloukas, Veronique Bataille, Jordana T. Bell

**Affiliations:** 1 Department of Twin Research and Genetic Epidemiology, King's College London, London, UK; 2 MRC London Institute of Medical Sciences, London, UK; 3 Institute of Clinical Sciences, Faculty of Medicine, Imperial College London, London, UK; 4 Department of Medical Sciences, Molecular Medicine and Science for Life Laboratory, Uppsala University, Uppsala, Sweden; 5 MRC Lifecourse Epidemiology Unit, University of Southampton, Southampton, UK; 6 Human Development and Health Academic Unit, Institute of Developmental Sciences, University of Southampton, Southampton, UK; 7 Epigenomic Medicine, Centre for Biological Sciences, Faculty of Environmental and Natural Sciences, University of Southampton, Southampton, UK; 8 William Harvey Research Institute, Queen Mary University of London, London, UK

**Keywords:** BMI, body mass index, CGI, CpG island, DMP, differentially methylated position, DMR, differentially methylated region, eQTL, expression quantitative loci, EWAS, epigenome-wide association study, FDR, false discovery rate, GWAS, genome-wide association studies, MAF, minor allele frequency, SNP, single nucleotide polymorphism

## Abstract

High nevus count is the strongest risk factor for melanoma, and although gene variants have been discovered for both traits, epigenetic variation is unexplored. We investigated 322 healthy human skin DNA methylomes associated with total body nevi count, incorporating genetic and transcriptomic variation. DNA methylation changes were identified at genes involved in melanocyte biology, such as *RAF1* (*P* = 1.2 × 10^−6^) and *CTC1* (region: *P* = 6.3 × 10^−4^), and other genes including *ARRDC1* (*P* = 3.1 × 10^−7^). A subset exhibited coordinated methylation and transcription changes within the same biopsy. The total analysis was also enriched for melanoma-associated DNA methylation variation (*P* = 6.33 × 10^−6^). In addition, we show that skin DNA methylation is associated in *cis* with known genome-wide association study single nucleotide polymorphisms for nevus count, at *PLA2G6* (*P* = 1.7 × 10^−49^) and *NID1* (*P* = 6.4 × 10^−14^), as well as melanoma risk, including in or near *MC1R*, *MX2,* and *TERT*/*CLPTM1L* (*P <* 1 × 10^−10^)*.* Our analysis using a uniquely large dataset comprising healthy skin DNA methylomes identified known and additional regulatory loci and pathways in nevi and melanoma biology. This integrative study improves our understanding of predisposition to nevi and their potential contribution to melanoma pathogenesis.

## Introduction

The total body number of melanocytic nevi is the strongest risk and predictive factor for melanoma in Caucasian populations (Gandini et al., 2005, Olsen et al., 2009). Melanoma is the most aggressive of skin tumors with an increasing incidence (Siegel et al., 2014). These malignancies arise from an existing benign nevus in 20% to 50% of cases (Haenssle et al., 2016, Purdue et al., 2005, Shitara et al., 2014, Weatherhead et al., 2007). The vast majority of nevi never progress to melanoma; however, nevi count is still a predisposition marker for melanoma arising de novo (Chang et al., 2009). Therefore, further understanding of the biology of nevi will give insights into the development and pathology of melanoma.

Typically, the number of nevi decrease after the age of 40; however, in individuals at high risk of melanoma, this loss of nevi is delayed, reflecting an altered senescence (Newton et al., 1993). Furthermore, a higher total body nevus count has also been associated with longer telomere length in blood in individuals from the TwinsUK cohort (Bataille et al., 2007). This link with senescence may indicate that the total numbers of nevi reflect differences in senescence pathways between individuals that can be detected in skin tissue where nevi are found. The usefulness of nevus counts as an intermediate phenotype to melanoma has already been shown in genome-wide association studies (GWAS), as common single nucleotide polymorphisms (SNPs) in the loci *PLA2G6* and *MTAP* were first associated with total body nevus count (Falchi et al., 2009, Nan et al., 2011) and then subsequently with melanoma risk (Barrett et al., 2011, Bishop et al., 2009). For nevus count, variants in *PLA2G6* were replicated across two studies that also identified additional associations in *NID1, c11orf74*, and *MTAP*. Although GWAS have identified genetic variation for nevi count and melanoma, the variance in nevus counts explained by these genes is low and no study has previously examined epigenetic variation in this context.

Here, we explore epigenome-wide DNA methylation variation in healthy human skin tissue in relation to total body nevus counts in 322 female individuals from the TwinsUK cohort. The focus of this study is on the potential to identify a predisposing DNA methylation signature in normal skin to the number of nevi and not the malignant changes occurring in melanocytes themselves. It has become increasingly acknowledged that crosstalk between all cells within the skin and melanocytes is important in the progression to melanoma (Kaur et al., 2016, Kim et al., 2013, Li et al., 2003, Shih et al., 1994). We investigated individual CpG differentially methylated positions (DMPs), as well as differentially methylated regions (DMRs) in healthy skin tissue, and corresponding gene expression changes within the same tissue. To study the potential interaction between genetic variants and DNA methylation, we examined the association between the skin DNA methylome and genetic variants previously associated with nevus count or melanoma risk by GWAS.

## Results

### The skin DNA methylome and its tissue layer specificity

**Figure 1:**
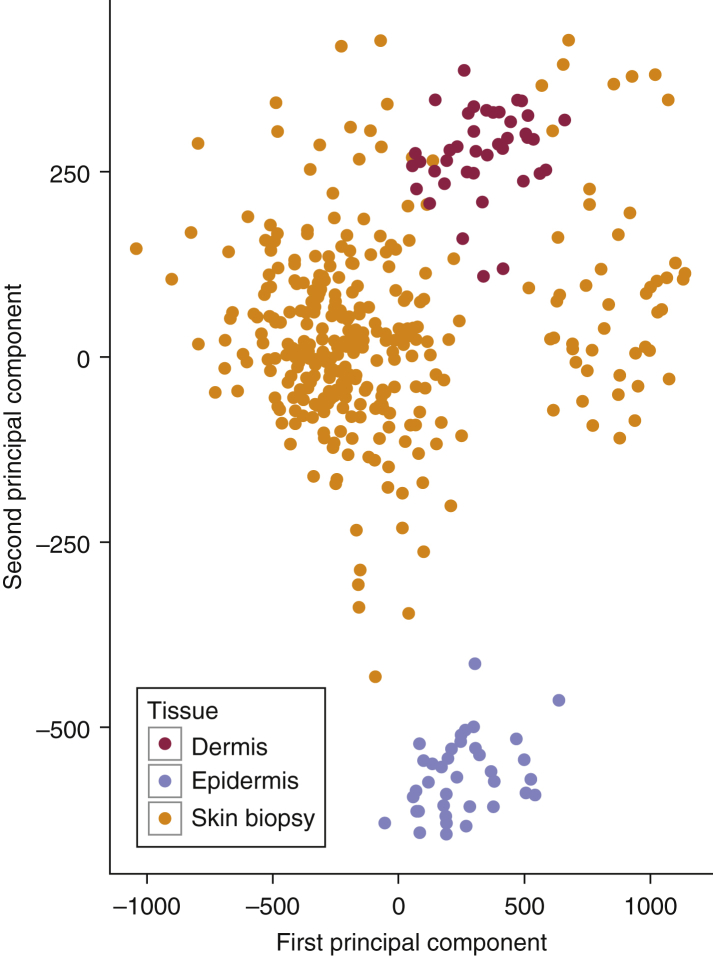
**Global DNA methylation profiles and skin tissue specificity.** First two principal components colored by layer specificity; red for dermal tissue, blue for epidermal tissue, and yellow for our data (see the legend).

As expected, because of the differing cell types in skin within the dermis and epidermis, these tissue layers harbor distinct DNA methylation profiles (Vandiver et al., 2015). In our study, skin tissue DNA was derived from a periumbilical punch biopsy (adipose tissue was removed from the biopsy before freezing) from 322 healthy female twins and profiled using the Infinium HumanMethylation450 BeadChip. To confirm which skin layer was represented in our biopsy sample, we compared our DNA methylation dataset with recently published DNA methylation profiles of mechanically separated epidermal (36 individuals) and dermal tissue (36 individuals) from Vandiver et al. (2015). Principal component analysis was performed on unadjusted DNA methylation profiles of the three groups of samples (dermis [n = 40], epidermis [n = 38], and our whole skin sample [n = 322]). The first two principal components explain 55.6% of the variance, capturing the skin layer specificity of the dermis and epidermis samples as previously shown (Vandiver et al., 2015). Our whole skin DNA methylation profiles cluster closely with the dermal layer DNA methylation profiles (Figure 1).

### Single CpG site differential skin DNA methylation in relation to total body nevus count

**Figure 2:**
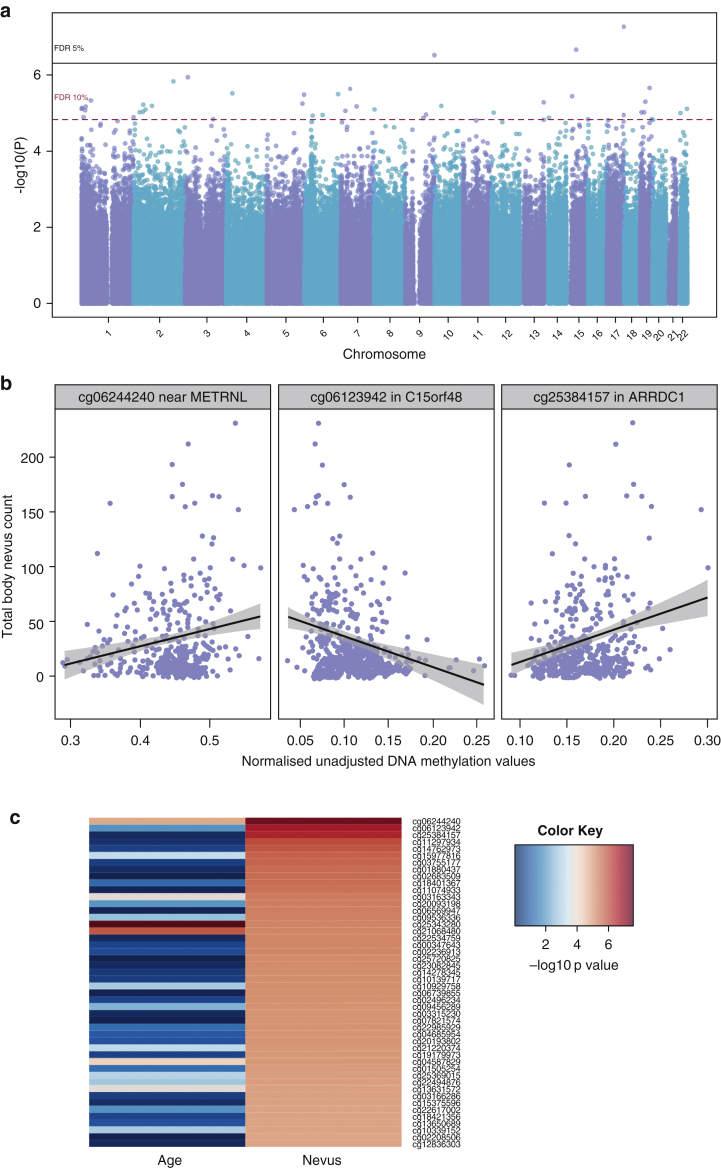
**Nevus count epigenome-wide results in 322 female individuals.** (**a**) Manhattan plot of the epigenome-wide association results in 322 female individuals, where each point represents the observed –log_10_*P*-value at a CpG site. (**b**) Panel plot depicting the direction of association at the three top-ranked signals for cg06244240 (left), cg06123942 (middle), and cg25384157 (right). Results are plotted using normalized unadjusted beta values per individual. The lines represent the linear correlation between DNA methylation and total body nevus count. (**c**) Heatmap of top 48 ranked n-DMPs colored by –log10 *P*-values of age association and nevus association. DMP, differentially methylated position.

We first explored evidence for differential skin DNA methylation associated with total body nevus count at the single CpG-site level across the genome in 322 healthy female twins. We fitted a linear mixed effects model regressing DNA methylation levels on fixed effects (age, body mass index [BMI], smoking status, chip, order on the chip, and bisulfite conversion efficiency) and random effects (family structure and zygosity). Three DMPs were identified to be significantly associated with total body nevus count (n-DMPs) at a false discovery rate (FDR) of 5% and a further 45 associations were observed at an FDR of 10% (Figure 2a, Table 1, Supplementary Table S1 online). The 48 n-DMPs are enriched for strong enhancers (ChromHMM state 4) in the normal human epidermal keratinocyte cell line derived from epidermal keratinocytes (*P =* 0.03) and for CpG island (CGI) shores (2 kb either side of the CGI, *P* = 0.04), while depleted for signals located in open sea genomic regions that are more than 4 kb beyond CGIs (*P* = 2.2 × 10^−3^).

**Table 1:** Most associated nevus differentially methylated positions (n-DMPs)

Rank	CpG	Position (hg19)	Associated gene	Location	CpG island	Beta	St. error	*P*-value	FDR
1	cg06244240	chr17: 81058948	–	–	Shore	0.0052	0.0009	5.52 × 10^−8^	5%
2	cg06123942	chr15: 45722795	*C15orf48*	5′UTR	Island	−0.0074	0.0014	2.20 × 10^−7^	5%
3	cg25384157	chr9: 140499131	*ARRDC1*	TSS1500	Shore	0.0063	0.0012	3.06 × 10^−7^	5%
4	cg11297934	chr3:12705868	*RAF1*	TSS200	Island	−0.0046	0.0009	1.16 × 10^−6^	10%
5	cg14762973	chr2:187714067	*ZSWIM2*	TSS200	Island	0.0069	0.0014	1.49 × 10^−6^	10%

Abbreviations: FDR, false discovery rate; TSS, transcription start site; UTR, untranslated region.

The strongest signals are shown Table 1 and Figure 2b. The most associated signal (cg06244240, *P* = 5.5 × 10^−8^) lies in a CGI shore approximately 6.5 kb downstream of *METRNL*, which is expressed in skin (Ushach et al., 2015) and is also involved in neural cell formation*.* The second ranked signal (cg06123942, *P* = 2.2 × 10^−7^) is in the 5′ CGI promoter of *C15orf48*, which displays reduced expression in squamous cell carcinoma (Freiberger et al., 2015). The third ranked signal (cg25384157, *P* = 3.1 × 10^−7^) is positively associated with the number of nevi and is in a CGI shore approximately 1.5 kb upstream of the transcription start site of *ARRDC1*, a negative regulator of the Notch signaling pathway (Puca et al., 2013). The CpG (cg11297934, *P* = 1.2 × 10^−6^) lies approximately 200 bp upstream of the transcription start site of proto-oncogene *RAF1* (also known as *CRAF)*, which is a member of the RAF family in the extracellular signal-regulated kinase/mitogen-activated protein kinase pathway that includes the key player *BRAF.*

Six of our top 10 n-DMPs did not show direct association with age (*P* = 0.05) including two of the top four results (*ARRDC1* and *RAF1*), with no n-DMP associations with age surpassing FDR 10% correction (Figure 2c). The remaining four signals did not show stronger evidence for association with age alone compared with nevus count. These results suggest that the majority of top-ranked n-DMPs are not directly associated with age.

### Regional differential skin DNA methylation associated with total body nevus count

We next aimed to identify DMRs, that is, small genomic regions that contain multiple CpG sites and show consistent directional association with total body nevus count (n-DMRs). DMRs have been found in general to be enriched for functionally relevant regions as well as for GWAS SNPs from the GWAS catalog (Ziller et al., 2013). We applied the BumpHunter (Jaffe et al., 2012) algorithm and identified 48 n-DMRs (*P* < 0.01) genome-wide (Table 2, Supplementary Table S2 online).

**Table 2:** Most associated nevus differentially methylated regions (n-DMRs)

Rank	Position (hg19)	Associated gene	Location	CpG island	Number of CpG sites	DNA methylation	*P-*value	Overview direction CpG sites
1	chr9:140499132–140500813	*ARRDC1*	TSS1500-Body	Island	7	+	2.5 × 10^−5^	+ + − − − + +
2	chr10:14647154–14647530	*FAM107B*	Body	Shore	3	+	2.5 × 10^−4^	+ + +
3	chr19:44285297–44285568	*KCNN4*	TSS200-1stExon	–	3	+	2.9 × 10^−4^	+ + +
4	chr17:8129997–8130356	*CTC1*	3′UTR	Shelf	3	–	6.3 × 10^−4^	− − −
5	chr15:26915414–26915752	*GABRB3*	Body	Island	3	–	8.3 × 10^−4^	− − −

Abbreviations: TSS, transcription start site; UTR, untranslated region.

**Figure 3:**
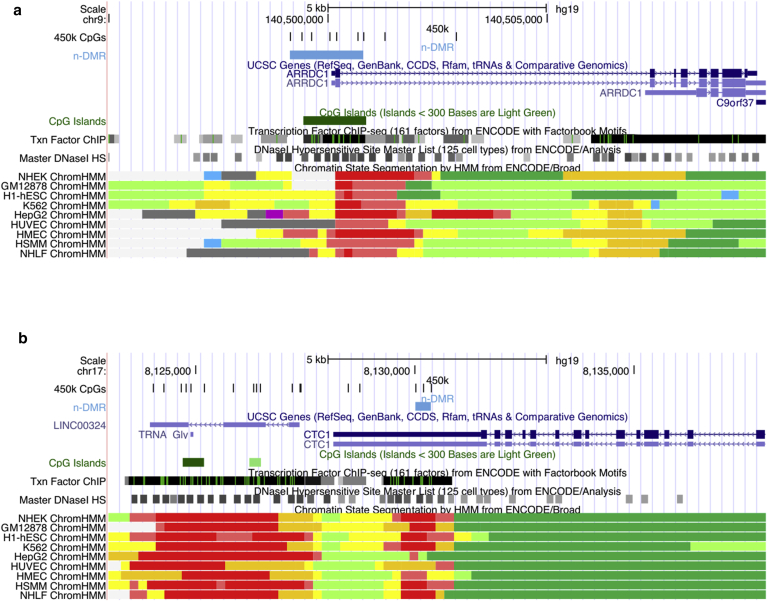
**Location of two top-ranked n-DMRs in the human genome.** Figures obtained from UCSC Genome browser, displaying position in the genome (hg19), CpG sites from HumanMethylation450 BeadChip, n-DMR (in light blue), RefSeq genes, CpG island, transcription factor ChIP data, DNase-I sensitivity sites, and ChromHMM genomic segmentation. (**a**) At *ARRDC1*. (**b**) At *CTC1*. DMR, differentially methylated region.

Subsequently, we examined the genomic context of the n-DMRs. The strongest signal (*P* = 6.8 × 10^−5^, Figure 3a) was observed overlapping the 5′ CGI promoter of *ARRDC1*. This n-DMR includes the third ranked FDR 5% individual CpG n-DMP (cg25384157). We also identified an n-DMR in *CTC1* that shows consistent negative association with total body nevus count amongst its three CpG sites (Figure 3b). It is in a region with active promoter evidence across multiple ENCODE cell types including normal human epidermal keratinocytes, which resides in the three prime untranslated region (3′ UTR) of *CTC1* but also 2 kb upstream of the lincRNA *LINC00324*. *CTC1* is a component of the CTS (CTC1, TEN1, and STN1) complex that has a pivotal role in protecting telomeres from degradation.

### Nevus differential methylation signatures reflected in gene expression changes

We then explored individual CpG sites in the 48 n-DMRs for association with gene expression levels using transcription profiles available in 248 individuals using the same skin biopsy as for DNA methylation profiling. The analysis focused on CpG sites that showed the same direction of effect in the n-DMR as in the single CpG site epigenome-wide association study (EWAS). We analyzed gene expression data at 36 genes for 27 n-DMRs, where the n-DMRs were either located in or were within 20 kb of the gene (Supplementary Table S3 online). Both DNA methylation and expression levels were adjusted for similar covariates and corresponding technical covariates and compared with Pearson correlation. At least one CpG site in 12 unique n-DMRs was shown to be correlated with the expression levels of 14 unique genes at nominal significance (*P* = 0.05, Supplementary Table S3). This included one of the three CpG sites forming the third ranked n-DMR in *KCNN4* (cg15977816, *r* = 0.19, *P* = 2.9 × 10^−3^). Strong n-DMRs that have more than one CpG site in the region correlated with one of more expression probes of the same gene include n-DMRs at *MED11*, *C14orf50*, *FAM64A*, *KRT86*, *TTC15*, and *C6orf27*.

### Nevus DNA methylation signature is enriched for melanoma-associated DNA methylation changes

Healthy tissue DNA methylomes can show risk-factor-related signatures also found in malignant tissue (Noreen et al., 2014). Furthermore, tumor methylation patterns have been shown to derive from the continuum of maturation states that are reflected in normal developmental stages (Oakes et al., 2016). We therefore examined the DNA methylome data of melanomas to see whether our identified n-DMP signature was enriched within this malignant tissue epigenome.

The most comprehensive melanoma DNA methylome study to date is a comparison between normal melanocytes from three donors and 27 metastatic melanoma DNA samples using methylated-CpG island recovery assay sequencing by Jin et al. (2015). They reported 3,113 regions that were hypermethylated in melanoma. Of these, 2,039 regions contained at least one CpG site profiled in our dataset and 13.4% (274 regions, containing 406 CpG sites) of these were identified as nominally significant with a positive association with total body nevus count. Within CGIs, which the methylated-CpG island recovery assay sequencing technique targets, we observed an enrichment of hypermethylated nominally significant CpG sites (Fisher’s *P* = 6.33 × 10^−6^). Another study, a genome-wide screen of promoter DNA methylation (24,103 RefSeq promoters) between normal skin, nevi, and advanced stage melanoma, identified four differentially methylated genes (Koga et al., 2009). At two of these genes, *THBS1* and *TNFRSF10D,* we identified a positive association between total body nevus count and promoter CpG methylation, in line with their findings of increased DNA methylation in advanced stage melanoma.

### Impact of GWAS SNPs associated with total body nevus count or melanoma risk on skin DNA methylation in *cis*

We next investigated the influence of genetic variants, previously associated in GWAS with nevus count or melanoma risk, on nearby DNA methylation levels in skin tissue for a subset of 283 individuals. We selected 4 SNPs previously associated with the number of cutaneous nevi (Falchi et al., 2009, Nan et al., 2011) and 23 SNPs associated with melanoma risk from the GWAS catalog (Welter et al., 2014), which fall within 100 kb of CpG sites available in our dataset. Mixed linear models were performed to test for association accounting for family structure and results are presented at a threshold of *P* < 1.0 × 10^−5^. This is comparable with a genome-wide cutoff at FDR 1%, as reported previously (*P* < 8.6 × 10^−4^), where all DNA methylation was assessed within 100 kb of common genetic variants (Grundberg et al., 2013).

**Figure 4:**
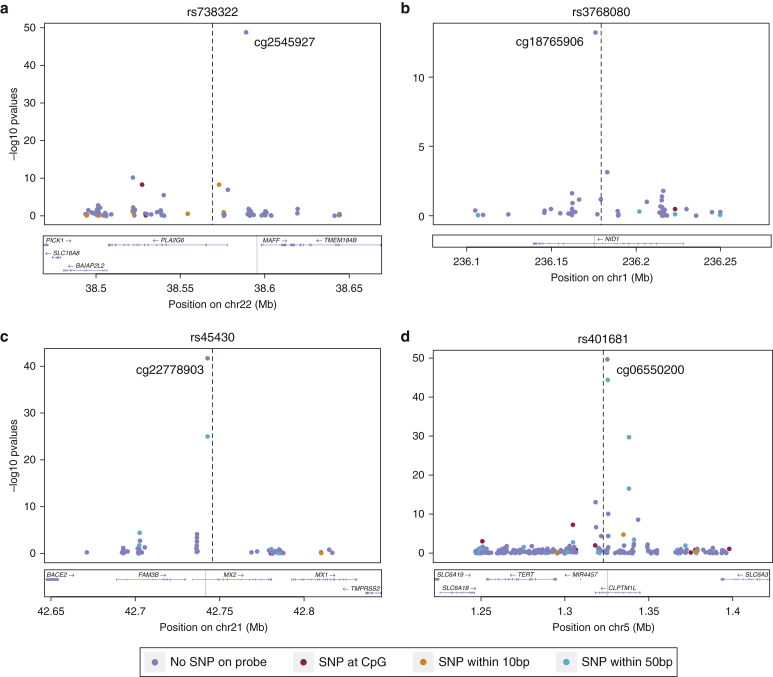
**GWAS SNPs for nevus count or melanoma risk versus DNA methylation in skin tissue in *cis*.** Regional plot of 100 kb flanking regions around the GWAS SNP of interest denoted as a striped black line. Each point is a CpG site with its *P-*value on the *y*-axis. The plots are annotated with a gene track from LocusZoom. Each point is colored according to the occurrence of genetic variants on the probe sequence (see the legend): (**a**) rs738322 identified for cutaneous nevi; (**b**) rs3768080 identified for cutaneous nevi; (**c**) rs45430 identified for melanoma risk; (**d**) rs401681 identified for melanoma risk. GWAS, genome-wide association studies; SNP, single nucleotide polymorphism.

Thirteen SNPs were significantly associated with DNA methylation changes in skin (*P* < 1 × 10^−5^, Table 3). These included three of the four SNPs identified by GWAS to be associated with total body nevus count: rs2284063 in intron of *PLA2G6* (cg25457927, *P* = 3.5 × 10^−38^), rs3768080 in intron of *NID1* (cg18765906, *P* = 6.4 × 10^−14^), and rs738322 in another intron of *PLA2G6* (cg25457927, *P* = 1.7 × 10^−49^). The results also included 10 SNPs previously identified for melanoma risk reported at *MC1R* (2 SNPs)*, MX2, TERT*/*CLPTM1L, PLA2G6, CASP8*, *ACTRT3*, *ASIP*, *CDC91L1*, and *ARNT/SETDB1/LASS2ANXA9/MCL1/CTSK* (Figure 4). Six of these CpG sites occurred in active promoters or enhancers in normal human epidermal keratinocytes (derived from epidermal keratinocytes) (Ernst et al., 2011). None of the CpG sites associated with these SNPs were epigenome-wide statistically significant n-DMPs or within n-DMRs from our EWAS analysis. However, nine nevus count CpG sites within these regions were associated with nevus count at nominal significance (*P* = 0.05), including the result at *PLA2G6*.

**Table 3:** Strongest CpG association per GWAS SNP

Trait	SNP	Position (hg19)	Reported genes	CpG	beta	*P*-value	Associated gene	Location	CpG island	Distance to SNP (kb)
Melanoma	rs401681	chr5:1322087	*TERT, CLPTM1L*	cg06550200	0.783	2.6 × 10^−50^	CLPTM1L	Body	–	−3.5
Cutaneous nevi	rs738322	chr22:38569006	*PLA2G6*	cg25457927	0.851	1.7 × 10^−49^	–	–	Shelf	−26.4
Melanoma	rs45430	chr21:42746081	*MX2*	cg22778903	−0.711	1.9 × 10^−42^	MX2	5′UTR	–	4.4
Melanoma	rs6001027	chr22:38545619	*PLA2G6*	cg25457927	0.843	1.3 × 10^−38^	–	–	Shelf	−49.8
Cutaneous nevi and Melanoma	rs2284063	chr22:38544298	*PLA2G6*	cg25457927	0.834	3.5 × 10^−38^	–	–	Shelf	−51.1
Melanoma	rs7412746	chr1:150860471	*ARNT, SETDB1, LASS2, ANXA9, MCL1, CTSK*	cg15448220	0.662	1.4 × 10^−32^	SETDB1	TSS1500	Shore	−37.4
Melanoma (2)	rs258322	chr16:89755903	*MC1R*	cg05714116	−0.728	5.5 × 10^−14^	CDK10	TSS1500	Shore	3.3
Cutaneous nevi	rs3768080	chr1:236179869	*NID1*	cg18765906	−0.374	6.4 × 10^−14^	NID1	Body	–	4.5
Melanoma	rs4785763	chr16:90066936	*MC1R*	cg08547343	−0.346	1.0 × 10^−9^	CENPBD1;AFG3L1	5′UTR; TSS200	Island	28.1
Melanoma	rs910873	chr20:33171772	*CDC91L1*	cg01901788	−0.441	1.8 × 10^−6^	MAP1LC3A	TSS1500	Shore	25.9
Melanoma	rs13097028	chr3:169464942	*ACTRT3*	cg27020690	−0.289	2.0 × 10^−6^	–	–	Island	−17.4
Melanoma	rs13016963	chr2:202162811	*CASP8*	cg24599065	−0.230	2.1 × 10^−6^	CASP10	3′UTR	–	69.0
Melanoma	rs228437	chr6:134898456	*ASIP*	cg24504307	−0.355	9.2 × 10^−6^	–	–	–	−64.7
Melanoma	rs3219090	chr1:226564691	*PARP1*	cg18764804	0.240	6.2 × 10^−4^	PARP1	TSS1500	Shore	−32.0
Melanoma	rs1031925	chr3:51379274	*DOCK3*	cg09456445	−0.315	8.5 × 10^−4^	DOCK3	3′UTR	Shore	−41.1
Melanoma	rs1722784	chr1:150961869	*ANXA9*	cg07479786	0.261	8.9 × 10^−4^	ANXA9	3′UTR	–	−6.0
Melanoma	rs16953002	chr16:54114824	*FTO*	cg01083598	−0.169	7.2 × 10^−3^	–	–	Island	−41.2
Melanoma	rs35390	chr5:33955326	*SLC45A2*	cg01990593	0.689	8.8 × 10^−3^	ADAMTS12	Body	Shore	65.0
Melanoma	rs1801516	chr11:108175462	*ATM*	cg08954307	−0.249	9.8 × 10^−3^	ATM	Body	–	−59.3
Cutaneous nevi	rs4636294	chr9:21747803	*MTAP*	cg03724238	−0.128	0.013	–	–	Island	51.1
Melanoma	rs4698934	chr4:106139387	*TET2*	cg08530497	−0.195	0.037	TET2	Body	–	−15.9
Melanoma	rs1847134	chr11:89005253	*TYR*	cg25941151	−0.153	0.041	TYR	TSS200	–	94.3
Melanoma (2)	rs7023329	chr9:21816528	*CDKN2A*	cg14548963	0.118	0.068	MTAP	Body	–	3.6
Melanoma (2)	rs1393350	chr11:89011046	*TYR*	cg03508346	−0.118	0.142	NOX4	3′UTR	–	−48.8
Melanoma	rs17119461	chr10:107516352	*NR*	cg18758405	−0.290	0.333	–	–	–	65.0
Melanoma	rs1889497	chr6:65432283	*EYS*	cg11999886	−0.026	0.760	EYS	Body	–	90.8

Abbreviations: GWAS, genome-wide association studies; SNP, single nucleotide polymorphism; TSS, transcription start site; UTR, untranslated region.

The 13 SNPs have not been previously reported as expression quantitative loci (eQTLs) using the same skin biopsy expression data (Grundberg et al., 2012). However, results from the Genotype-Tissue Expression Project (GTEx Consortium, 2015) datasets show that eight of these SNPs are eQTLs in either skin tissue (sun or not sun exposed) and/or transformed fibroblasts. These GTEx eQTLs were associated with the expression of 16 genes in total, and at 7 of these we identified DNA methylation variation associated with the same SNP: *CASP8* (rs1301693), *MAFF, PLA2G6, TMEM184B,* and *BAIAP2L2* (rs2284063, rs738322, and rs6001027), *SPATA33* (rs258322), *MX2* (rs45430), and *CDK10* (rs4785763).

## Discussion

To our knowledge, this is the first study to explore epigenome-wide DNA methylation in the largest healthy human skin tissue dataset to date, in relation to the number of nevi, the strongest risk factor of melanoma. This analysis adds an additional layer to our current understanding of the genomic biology of nevi development on top of that previously obtained via GWAS. We used normal skin biopsies of 322 healthy female twins and showed that these biopsies represent mostly homogenous dermis cells due to their distinct epigenetic signature. We identified DNA methylation variation associated with total body nevus count at, to our knowledge, previously unreported genes and also at known genes in nevi formation or melanoma, both at single CpGs and regions. These top-ranked results are significantly enriched for strong enhancers in normal human epidermal keratinocyte cell lines as well as for CGI shores, regions identified as more dynamic and functional in both cancer (Irizarry et al., 2009) and stem cell reprogramming (Doi et al., 2009). Approximately half of the regional epigenetic changes tested also had corresponding gene expression differences in the same biopsy. Finally, we identified DNA methylation variation in *cis* associated with known GWAS SNPs for both nevi number and melanoma risk.

We identified many genomic loci that exhibited differential methylation associated with total body nevus count that were highly relevant to melanocyte biology and cancer. Among these is the n-DMR at *CTC1*, involved in telomere maintenance and associated with telomere length (Mangino et al., 2012). Telomere length has been positively associated with high nevus count and melanoma risk (Bataille et al., 2007), as well as via a genetic score analysis from seven telomere length associated SNPs (Iles et al., 2014). This suggests that telomeres within healthy skin may already be longer in high nevus count individuals as a similar rate of age-dependent telomere length attrition has been shown between leukocytes and skin (Daniali et al., 2013), and supports previous work in melanoma biology. It may also explain why individuals with multiple atypical nevi are relatively protected against photo-aging, due to potential reduced skin senescence.

An important molecular pathway highlighted, due to differential DNA methylation in *RAF1,* is the mitogen-activated protein kinase/extracellular signal-regulated kinase pathway that includes *BRAF*. *BRAF* mutations are found in approximately 50% of all melanoma tumors and are of significant clinical utility as major therapeutic decisions are made according to the presence or absence of mutations in this gene (Flaherty et al., 2010). Somatic mutations in the oncogene *RAF1* are remarkably rare in human cancers (Emuss et al., 2005); however, in *BRAF* negative melanomas, targeting *RAF1* leads to apoptosis. Therefore, the significant identification that this pathway is altered via DNA methylation could lead to the re-evaluation of using mutational status alone in advanced melanoma treatment decisions (Heyn and Esteller, 2012).

Another result of note is *ARRDC1*, part of the highly conserved cell signaling NOTCH pathway. This pathway is pivotal in cell-fate determination across many organ systems in embryogenesis and also tissue maintenance in adults. Expression of *Notch1* is low or not detectable in melanocytes as well as nevi; however, higher expression is associated with their transformation to melanoma (Massi et al., 2006, Pinnix et al., 2009). *ARRDC1* is necessary for Itch E3 ubiquitin ligase-mediated NOTCH receptor degradation (Puca et al., 2013).

We also found that our nevi signature was enriched for DNA methylation changes previously identified in melanoma (Jin et al., 2015). This may indicate a priming or predisposition to melanoma of normal skin with more nevi. We then investigated the effect of 26 unique genetic variants, previously associated by GWAS with nevus number (4 SNPs) or melanoma risk (23 SNPs), on DNA methylation within 100 kb (Falchi et al., 2009, Nan et al., 2011, Welter et al., 2014). Half of these SNPs were significantly associated with DNA methylation changes in skin tissue. For all three GWAS SNPs reported for *PLA2G6,* which is associated with both nevus numbers and melanoma, eQTLs have been reported in skin and/or transformed fibroblasts impacting on the expression of *PLA2G6, MAFF, TMEM184B,* and *BAIAP2L2.* All of these genes harbored SNP-associated DNA methylation variation in our study. This highlights the usefulness of integrating DNA methylation analysis with robust GWAS discoveries to identify functional pathways underlying strong genetic signals. Furthermore, we identified GWAS SNP-associated DNA methylation variation, not only in genes with known eQTLs, but also in novel genes.

Compared with other studies, our sample is much larger than previous skin DNA methylation studies (Roberson et al., 2012, Rodríguez et al., 2014, Vandiver et al., 2015), and we have profiled only women, who are known to have different distributions of nevi on the body compared with men (Autier et al., 2004) as well as sex-specific differences in DNA methylation (Singmann et al., 2015). In addition, this study benefits from the extensive data for nevus counts collected by trained dermatology nurses of healthy individuals. In contrast to blood-based EWAS, this study also highlights the strength of investigating the phenotype-appropriate tissue to help understand biological pathways implicated in disease.

To conclude, this study based on skin biopsies of 322 healthy female Caucasians using DNA methylome, transcriptome, and GWAS data from the same individuals identified DNA methylation differences associated with the number of nevi. We identified pathways and genes that add to our current understanding of the biology of the number of nevi and the pathology of melanoma. These data open up avenues for future exploration in skin, not only why some individuals have higher numbers of nevi but how this phenotype contributes to melanoma risk.

## Materials and Methods

### Sample selection

Detailed information regarding nevus count was obtained from twins registered with the TwinsUK Adult Twin Registry. The twins in this registry are not selected for diseases and were similar in means and ranges of quantitative phenotypes to an age-matched population in the UK (Andrew et al., 2001). Written informed consent from all subjects was obtained in accordance with Guy’s and St Thomas’ National Health Service Foundation Trust Ethics Committee (EC04/015—15-Mar-04). The selected individuals did not have a personal medical history of malignant melanoma or other skin cancers obtained through record linkage with the National Cancer Registry at the Office for National Statistics. The examination was performed by trained research nurses following a standardized and reproducible nevus count protocol as described previously (Bataille et al., 2000). Total body nevus count was the sum of all nevi >2 mm across 17 body sites.

DNA methylation data profiled from skin tissue were available for 322 female twins with a mean age of 59 years, including 25 monozygotic twin pairs, 64 dizygotic twin pairs, and 144 unrelated individuals of European descent. Punch biopsies (8 mm) were taken from a relatively photo-protected area adjacent and inferior to the umbilicus and were mechanically dissected for skin tissue removing the fat layer before freezing. Written informed consent from all study subjects was obtained and the procedures were in accordance with the ethical standards of the St. Thomas’ Research Ethics Committee (REC reference 07/H0802/84). The skin biopsy samples were obtained within 6.5 and 11.9 years after nevus count with a mean of 9.7 years (Supplementary Figure S1 online).

### Genome-wide DNA methylation profiles

Genome-wide DNA methylation profiles were obtained from 326 bisulfite-converted skin tissue DNA samples assayed by Illumina Infinium HumanMethylation450 BeadChip (Illumnia, San Diego, CA). DNA methylation levels were denoted as betas: the ratio of intensity signal from the methylated probes over the sum of intensity signals from both unmethylated and methylated probes. Multiple measurements of analytical quality were applied. Probes were removed for the main analyses that failed detection in at least one sample or had a bead count less than 3 in more than 1% of the samples (n = 18,208), had a 50 bp probe sequence that aligned to multiple locations in the genome (n = 17,764), harbored common genetic variants (minor allele frequency [MAF] ≥ 1%) within 10 bp on the probe at the interrogated CpG site (15,827), or contained variants at any MAF within 10 bp at the interrogated CpG site (11,236), and were located on the sex chromosomes (n = 11,650). Probes with genetic variants within the 50 bp of the CpG, but not within 10 bp, were not excluded for GWAS SNP analyses but were highlighted as such in the figures and accompanying text.

Individuals were verified using the 57 autosomal SNP probes included as control probes on the BeadChip. Overall intensity signal as well as bisulfite conversion efficiency was assessed and the data were inspected visually for outliers using beta density plots, boxplots, and imprinted regions (using the R package wateRmelon [Pidsley et al., 2013]). Four individual samples were excluded based on low mean intensity signals. The remaining 322 samples were normalized using the beta mixture quantile dilation method to correct for probe-type bias (Teschendorff et al., 2013). Principal component analysis was performed on beta mixture quantile dilation normalized beta values that were standardized (N(0,1)) at each probe. The first three principal components, which when combined explain 36% of the total variance, were assessed for associations with possible confounders for DNA methylation data including BeadChip, position on the BeadChip, age, smoking status, BMI, and bisulfite conversion efficiency. Strongly significant associations (*P* < 1 × 10^−20^) were identified with BeadChip and bisulfite conversion efficiency.

### Gene expression profiles

Gene expression profiles were obtained of the same skin tissue biopsies that were profiled for DNA methylation for 248 individuals from the MuTHER project (Multiple Tissue Human Expression Resource) as previously described (Grundberg et al., 2012). In short, punch biopsies (8 mm) were taken from a photo-protected area adjacent and inferior to the umbilicus of which skin tissue was dissected. RNA was extracted and expression profiling was performed using Illumina Human HT-12 V3 BeadChips. Probes with less than three beads present were excluded and log_2_-transformed expression signals were normalized separately per tissue, with quantile normalization of the replicates of each individual followed by quantile normalization across all individuals.

### Genotypes

Genotype data of 283 individuals were available from the TwinsUK dataset as previously described (Small et al., 2011), genotyped using a combination of Illumina HumanHap300, HumanHap610Q, 1M-Duo, or 1.2M-Duo custom arrays. Imputation was done based on 1000 Genomes data phase 3. Quality control measures included minimum genotyping rate (>95%), Hardy-Weinberg equilibrium (*P* > 10^−6^), minimum MAF (>1%), and imputation quality score >0.5 for GWAS catalog SNPs. This subset of individuals included only individuals of Caucasian ancestry.

### Statistical analysis

DMPs associated with total body nevus count were identified using a linear mixed effects model fitted on the normalized beta values per probe (N(0,1)) with total body nevus count, age, BMI, smoking, bead chip, position on the bead chip, and bisulfite conversion efficiency as fixed effects, and family and zygosity as random effects. This model was compared with a null model that excluded total body nevus count by analysis of variance. Results were considered genome-wide significant if they surpassed an FDR threshold of 5% and considered suggestive when surpassing an FDR of 10%, estimated using “qvalue” in R.

Nevus count DMPs were tested for association with age using a linear mixed effects model fitted on the normalized beta values per probe (N(0,1)) with age, BMI, smoking status, BeadChip, position on the BeadChip, and bisulfite conversion efficiency as fixed effects, and family and zygosity as random effects. This was compared with a null model without age by analysis of variance to test for significance.

Small DMRs associated with nevus count were identified using R package “Bumphunter” (Jaffe et al., 2012). Regions required at least three consecutive probes with a maximum gap of 500 bp between each probe. DNA methylation was adjusted for covariates described previously for DMP analysis before using Bumphunter. Nevus count DMRs were considered with a *P*-value <0.01 estimated based on 1,000 permutations.

Gene expression analysis of the top-ranked DMRs was performed in a sample of 248 individuals using the same skin tissue biopsy used for DNA methylation profiling. A linear mixed model was fitted on the expression data with age, BMI, smoking status, batch, and concentration (fixed effects) and family and zygosity (random effects) as well as the linear mixed model fitted for DNA methylation data described previously for n-DMP analysis. The residuals from both models were compared with Pearson correlation.

Genotype variation analyses using GWAS SNPs were performed in a sample of 283 individuals using genome-wide efficient mixed model association to account for family structure present in the data and adjusted DNA methylation levels for fixed covariates used in the n-DMP analysis (age, BMI, smoking status, BeadChip, position on the BeadChip, and bisulfite conversion efficiency).

## Availability of Data and Materials

The DNA methylation data of the 322 individuals for this article are in the Gene Expression Omnibus (GEO) with accession code GSE90124.

## ORCID

Leonie Roos: http://orcid.org/0000-0001-7885-2105

## Conflict of Interest

The authors state no conflict of interest.
