# Supplementary material for: Higher Nevus Count Exhibits a Distinct DNA Methylation Signature in Healthy Human Skin: Implications for Melanoma
Source: J Invest Dermatol. 2017 Apr;137(4):910–20. doi: 10.1016/j.jid.2016.11.029 (PMC5754330; doi:10.1016/j.jid.2016.11.029)
Supplement: Supplementary Figure S1 and Supplementary Tables S1–S3 [file mmc1.pdf]

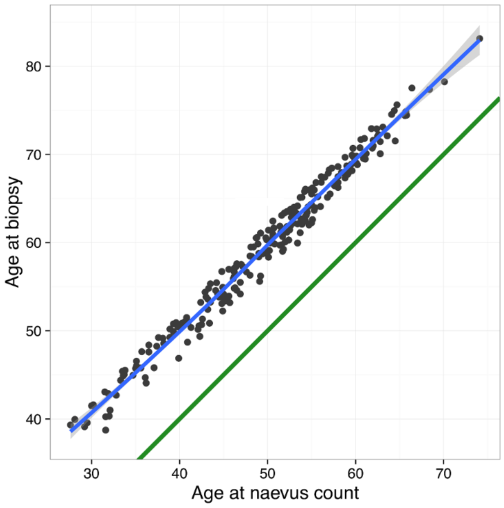

Figure S1: Age at naevus examination.
Correlation of chronological ages between naevus examination and skin punch biopsy. The blue line is the true least squares regression fit whereas the green line is a perfect age match.

Table S1. Most associated naevus count differential methylation positions (nDMPs) at FDR 10% or lower.

Rank	CpG	Position (hg10)	Associated gene	Location	CpG Island	Beta	St. Error	P value
1	cg06244240	chr17:81058949	-	-	Shore	0.0052	0.0009	5.52E-08
2	cg06123942	chr15:45722796	C15orf48	5'UTR;1stExon	Island	-0.0074	0.0014	2.20E-07
3	cg25384157	chr9:140499132	ARRDC1	TSS1500	Shore	0.0063	0.0012	3.06E-07
4	cg11297934	chr3:12705869	RAF1	TSS200	Island	-0.0046	0.0009	1.16E-06
5	cg14762973	chr2:187714068	ZSWIM2	TSS200	Island	0.0069	0.0014	1.49E-06
6	cg15977816	chr19:44285297	KCNN4	5'UTR;1stExon	-	0.0061	0.0013	2.23E-06
7	cg03755177	chr7:44349389	CAMK2B	Body	Island	-0.0066	0.0014	2.34E-06
8	cg01880437	chr4:26321873	RBPJ	TSS1500	Island	-0.0057	0.0012	3.06E-06
9	cg02683509	chr6:156950855	-	-	Shore	0.0059	0.0012	3.22E-06
10	cg18401367	chr5:176107201	-	-	Island	-0.0063	0.0013	3.32E-06
11	cg11074933	chr15:26915414	GABRB3	Body	Island	-0.0063	0.0013	3.65E-06
12	cg03163343	chr1:45118395	-	-	Shore	0.0049	0.0010	4.72E-06
13	cg20093198	chr19:24182837	-	-	-	0.0053	0.0011	5.11E-06
14	cg06569947	chr13:114126889	DCUN1D2	Body	-	-0.0060	0.0013	5.28E-06
15	cg09536336	chr5:168439657	SLIT3	Body	-	0.0053	0.0012	5.70E-06
16	cg25343280	chr2:44314198	-	-	Shore	0.0055	0.0012	6.06E-06
17	cg21068480	chr2:85980500	ATOH8	TSS1500	Island	0.0057	0.0012	6.52E-06
18	cg22534759	chr10:32403029	-	-	Shelf	0.0044	0.0010	6.53E-06
19	cg00347643	chr7:75957202	YWHAG	3'UTR	Shore	-0.0053	0.0011	6.75E-06
20	cg02236913	chr1:20005598	HTR6	Body	Island	0.0059	0.0013	6.81E-06
21	cg25720825	chr1:2849682	-	-	Shore	0.0053	0.0012	7.46E-06
22	cg23082845	chr1:1159282	SDF4	Body	Island	0.0056	0.0012	7.76E-06
23	cg14278345	chr22:50451054	IL17REL	5'UTR;1stExon	Shore	0.0046	0.0010	7.85E-06

24	cg10139717	chr8:2363092	-	-	-	-0.0056	0.0012	8.11E-06
25	cg10929758	chr2:54857270	SPTBN1	Body	Shore	0.0051	0.0011	8.17E-06
26	cg06739855	chr1:17240204	-	-	Island	-0.0057	0.0013	8.53E-06
27	cg02496234	chr7:20826128	SP8	5'UTR	Shore	0.0061	0.0013	8.82E-06
28	cg09456289	chr2:43038399	-	-	Island	-0.0063	0.0014	9.36E-06
29	cg03315230	chr2:27346938	ABHD1	Body	Shore	0.0045	0.0010	9.61E-06
30	cg07821574	chr19:18208505	MAST3	TSS200	Shore	-0.0042	0.0009	9.66E-06
31	cg22985929	chr19:6227536	MLLT1	Body	Shelf	0.0050	0.0011	9.71E-06
32	cg04685954	chr12:10515574	-	-	-	0.0043	0.0010	9.82E-06
33	cg20193802	chr22:19709696	SEPT5;GP1BB	Body;TSS1500	Island	0.0050	0.0011	1.01E-05
34	cg21220374	chr9:100396777	TSTD2;NCBP1	TSS1500;Body	Shore	0.0058	0.0013	1.11E-05
35	cg19179973	chr6:83775554	UBE2CBP	TSS200	Island	0.0040	0.0009	1.13E-05
36	cg04587829	chr17:80692947	FN3K	TSS1500	Shore	0.0048	0.0011	1.14E-05
37	cg01505254	chr6:36853867	C6orf89	Body	Island	-0.0053	0.0012	1.18E-05
38	cg25369015	chr1:9599256	SLC25A33	TSS1500	Shore	-0.0063	0.0014	1.29E-05
39	cg22494876	chr1:247618161	-	-	Shelf	0.0060	0.0014	1.30E-05
40	cg13631572	chr14:24803903	ADCY4	TSS200	Island	-0.0058	0.0013	1.35E-05
41	cg03166286	chr9:88555512	NAA35	TSS1500	Shore	0.0058	0.0013	1.36E-05
42	cg15375596	chr15:99434736	IGF1R	Body	Island	0.0053	0.0012	1.41E-05
43	cg22617002	chr13:112275633	-	-	Island	0.0054	0.0012	1.45E-05
44	cg18421356	chr3:134204114	ANAPC13;CEP63	5'UTR;TSS1500	Shore	-0.0050	0.0011	1.46E-05
45	cg13650689	chr19:54618596	PRPF31;TFPT	TSS200;Body	Shore	-0.0049	0.0011	1.48E-05
46	cg10339152	chr20:749452	C20orf54	TSS1500	-	0.0041	0.0009	1.53E-05
47	cg02208506	chr17:4634804	MED11	1stExon	Island	-0.0055	0.0013	1.55E-05
48	cg12836303	chr11:63756920	OTUB1	Body	Shelf	0.0059	0.0013	1.55E-05

Table S2. Most associated naevus count differential methylation regions (nDMRs) with *P* value < 0.01.

Rank	Position (hg 19)	Associated Gene	Location	CpG Island	Number of CpG sites	DNA methylation	*P* value	Overview direction CpG sites
1	chr9:140499132-140500813	ARRDC1	TSS1500-Body	Island	7	+	2.6E-05	+ + - - - + +
2	chr10:14647154-14647530	FAM107B	Body	Shore	3	+	2.5E-04	+ + +
3	chr19:44285297-44285568	KCNN4	TSS200-1stExon	-	3	+	2.9E-04	+ + +
4	chr17:8129997-8130356	CTC1	3'UTR	Shelf	3	-	6.3E-04	- - -
5	chr15:26915414-26915752	GABRB3	Body	Island	3	-	8.3E-04	- - -
6	chr1:165513318-165513343	LOC400794; LRRC52	Body;TSS200	-	3	+	9.6E-04	+ + +
7	chr7:44349389-44349389	CAMK2B	Body	Island	1	-	1.2E-03	-
8	chr8:130995990-130996123	-	-	Island	3	-	1.2E-03	- - -
9	chr13:114126889-114126889	DCUN1D2	Body	-	1	-	1.3E-03	-
10	chr11:64739320-64739343	-	-	Island	3	-	1.8E-03	- - -
11	chr10:102279455-102279694	SEC31B	TSS200-5'UTR	Island	3	-	2.2E-03	- - -

12	chr19:43968133-43968495	LYPD3	Body	Island	2	-	2.2E-03	- -
13	chr2:4600947-4601053	-	-	-	3	+	2.5E-03	+ + +
14	chr20:5485270-5485294	LOC149837	TSS200	-	3	-	2.6E-03	- - -
15	chr7:152063901-152063974	MLL3	Body	Island	3	-	2.6E-03	- - -
16	chr7:98030324-98030641	BAIAP2L1	TSS1500-TSS200	Shore	4	+	2.8E-03	+ + - +
17	chr1:151693222-151693261	C1orf230	TSS1500	Shore	2	-	2.8E-03	- -
18	chr1:17240204-17240204	-	-	Island	1	-	2.9E-03	-
19	chr8:82633130-82633568	ZFAND1	TSS200-Body	Island	4	-	3.0E-03	- + - -
20	chr11:102576469-102576508	MMP27	TSS200	-	2	+	3.4E-03	+ +
21	chr2:85980500-85980500	ATOH8	TSS1500	Island	1	+	3.4E-03	+
22	chr1:27729801-27729992	-	-	-	2	+	3.5E-03	+ +
23	chr14:38091400-38091470	-	-	Shore	2	-	3.8E-03	- -
24	chr4:26321873-26321873	RBPJ	TSS1500	Island	1	-	3.8E-03	-
25	chr12:1905735-	CACNA2D4	Body	Island	2	+	3.8E-03	+ +

	1906097							
26	chr18:56296449-56296607	ALPK2	TSS1500	-	4	+	3.9E-03	+ + + +
27	chr22:45598944-45599059	C22orf9	Body	Island	2	+	3.9E-03	+ +
28	chr1:9599256-9599276	SLC25A33	TSS1500	Shore	2	-	4.2E-03	- -
29	chr7:105331648-105331690	ATXN7L1	Body	-	2	-	4.2E-03	- -
30	chr16:2502146-2502146	CCNF	Body	-	1	-	4.4E-03	-
31	chr1:1159282-1159282	SDF4;SDF4	Body;Body	Island	1	+	4.8E-03	+
32	chr1:43770557-43770707	TIE1	Body	Island	2	-	5.1E-03	- -
33	chr17:4634804-4634804	MED11	1stExon	Island	1	-	5.5E-03	-
34	chr1:162039224-162039224	NOS1AP	TSS1500	Shore	1	-	5.6E-03	-
35	chr14:65016591-65016602	C14orf50	TSS200	Island	3	+	5.9E-03	+ + +
36	chr12:54132255-54132568	-	-	Shore	3	-	6.4E-03	- - -
37	chr17:6347533-6347533	FAM64A	TSS1500	Island	1	-	6.7E-03	-
38	chr2:44314198-44314198	-	-	Shore	1	+	6.9E-03	+

39	chr16:28565199-28565206	CCDC101	TSS200	Island	2	-	7.2E-03	- -
40	chr4:55092375-55092556	-	-	Shore	2	+	7.4E-03	+ +
41	chr7:152456579-152456597	ACTR3B	TSS1500	Shore	2	+	8.2E-03	+ +
42	chr22:39712694-39712730	RPL3;SNORD83A;RPL3	Body;TSS1500;Body	Shelf	2	+	8.4E-03	+ +
43	chr9:100396777-100396777	TSTD2;NCBP1	TSS1500;Body	Shore	1	+	8.9E-03	+
44	chr17:78865087-78866235	RPTOR	Body	Shore	8	+	8.9E-03	+ + + + + + + +
45	chr18:43548144-43548144	KIAA1632	TSS1500	Shore	1	+	9.1E-03	+
46	chr12:52695412-52695515	KRT86	TSS1500-TSS200	Shore	2	-	9.4E-03	- -
47	chr2:3471345-3471345	TTC15	Body	-	1	+	9.7E-03	+
48	chr6:31743928-31743952	C6orf27	Body	-	2	+	9.8E-03	+ +

Table S3. Naevus count differentially methylated regions vs expression levels.

Rank	Position (hg 19)	Associated Gene	Location	DNA methylation	Transcript	Associated Gene	Correlation	P value
3	chr19:44285297-44285568	KCNN4	TSS200-1stExon	+	ILMN_1709937	KCNN4	0.19	2.9E-03
13	chr2:4600947-4601053	-	-	+	ILMN_1692706	DCUN1D2	-0.14	0.033
17	chr1:151693222-151693261	C1orf230	TSS1500	-	ILMN_1681234	TNRC4	0.13	0.037
19	chr8:82633130-82633568	ZFAND1	TSS200-Body	-	ILMN_2087989	ZFAND1	0.14	0.025
33	chr17:4634804-4634804	MED11	1stExon	-	ILMN_1762639	MED11	0.19	2.6E-03
35	chr14:65016591-65016602	C14orf50	TSS200	+	ILMN_2153916	HSPA2	-0.13	0.047
37	chr17:6347533-6347533	FAM64A	TSS1500	-	ILMN_2415292	AIPL1	-0.18	5.5E-03
39	chr16:28565199-28565206	CCDC101	TSS200	-	ILMN_1810560	P8	0.13	0.035
42	chr22:39712694-39712730	RPL3;SNORD83A;RPL3	Body;TSS1500;Body	+	ILMN_1653927	SNORD83A	0.13	0.034
46	chr12:52695412-52695515	KRT86	TSS1500-TSS200	-	ILMN_1677586	KRT83	0.13	0.035
46	chr12:52695412-52695515	KRT86	TSS1500-TSS200	-	ILMN_1801442	KRT81	-0.19	3.2E-03
47	chr2:3471345-	TTC15	Body	+	ILMN_1693317	TTC15	-0.14	0.024

	3471345							
48	chr6:31743928-31743952	C6orf27	Body	+	ILMN_1696601	VARS	-0.18	5.5E-03
48	chr6:31743928-31743952	C6orf27	Body	+	ILMN_1755507	VARS	0.14	0.025
48	chr6:31743928-31743952	C6orf27	Body	+	ILMN_1810834	MSH5	-0.16	0.014
